# Patient Engagement Practices in Clinical Research among Patient Groups, Industry, and Academia in the United States: A Survey

**DOI:** 10.1371/journal.pone.0140232

**Published:** 2015-10-14

**Authors:** Sophia K. Smith, Wendy Selig, Matthew Harker, Jamie N. Roberts, Sharon Hesterlee, David Leventhal, Richard Klein, Bray Patrick-Lake, Amy P. Abernethy

**Affiliations:** 1 School of Nursing, Duke University, Durham, NC, United States of America; 2 WS Collaborative, McLean, VA, United States of America; 3 Clinical Trials Transformation Initiative, Duke Clinical Research Institute, Durham, NC, United States of America; 4 Parent Project Muscular Dystrophy, Hackensack, NJ, United States of America; 5 Pfizer Worldwide Research and Development, Groton, CT, United States of America; 6 U.S. Food and Drug Administration, Silver Spring, MD, United States of America; 7 Flatiron Health, New York, NY, United States of America; Renal Division, Peking University First Hospital, CHINA

## Abstract

**Objective:**

Patient-centered clinical trial design and execution is becoming increasingly important. No best practice guidelines exist despite a key stakeholder declaration to create more effective engagement models. This study aims to gain a better understanding of attitudes and practices for engaging patient groups so that actionable recommendations may be developed.

**Methods:**

Individuals from industry, academic institutions, and patient groups were identified through Clinical Trials Transformation Initiative and Drug Information Association rosters and mailing lists. Objectives, practices, and perceived barriers related to engaging patient groups in the planning, conduct, and interpretation of clinical trials were reported in an online survey. Descriptive and inferential statistical analysis of survey data followed a literature review to inform survey questions.

**Results:**

Survey respondents (n = 179) valued the importance of involving patient groups in research; however, patient group respondents valued their contributions to research protocol development, funding acquisition, and interpretation of study results more highly than those contributions were valued by industry and academic respondents (all p < .001). Patient group respondents placed higher value in open communications, clear expectations, and detailed contract execution than did non–patient group respondents (all p < .05). Industry and academic respondents more often cited internal bureaucratic processes and reluctance to share information as engagement barriers than did patient group respondents (all p < .01). Patient groups reported that a lack of transparency and understanding of the benefits of collaboration on the part of industry and academia were greater barriers than did non–patient group respondents (all p< .01).

**Conclusions:**

Despite reported similarities among approaches to engagement by the three stakeholder groups, key differences exist in perceived barriers and benefits to partnering with patient groups among the sectors studied. This recognition could inform the development of best practices for patient-centered clinical trial design and execution. Additional research is needed to define and optimize key success factors.

## Introduction

Tens of thousands of patient groups and voluntary health organizations exist in the United States [1]. This sector is large, diverse, continually evolving, and therefore difficult to track. Some organizations are well-established nonprofits (e.g., American Cancer Society, March of Dimes); others are relatively new. Some groups focus on diseases that affect large numbers of people, such as diabetes and cancer. Others target rare or “orphan” diseases such as cystic fibrosis [2]. Today, patient groups are facilitating clinical research by moving beyond traditional roles of patient recruitment and education to influencing funding decisions, informing research priorities, collaborating with industry, and contributing money for research and patient care [3].

Patient-powered registries and research networks developed by patient organizations are rapidly evolving as a means to contribute to research that leads to significant improvements in patient engagement, care, and health [4,5]. Patient groups are establishing tools and resources to fulfill unmet needs and provide more sophisticated ways to tailor patient group engagement in the research process. Examples include the Fox Trial Finder for Parkinson’s treatment acceleration with a novel volunteer/patient engagement model [6]; JDRF trials connection for type 1 diabetes [7]; Crohnology.com for sharing experiences with Crohn’s and colitis [8]; and other examples of proactive guidance on benefit/risk frameworks [9] and drug development roundtables [10]. Although no single formula exists for how best to engage with patient groups, two reports by the Institute of Medicine and the Clinical and Translational Science Awards task force on community engagement provide useful guidance for partnering with such organizations [11,12]. These reports stress the need for “meaningful engagement” in setting research priorities, governance of comparative effectiveness research programs, framing of research questions and protocols, monitoring of trials, and interpreting and disseminating results.

While there is wide agreement on the importance of incorporating the “voice of the patient” into the clinical research continuum, disagreements remain about the goals of patient engagement beyond the elements of recruitment and retention (a common motivation in industry) and whether there is a funding/sponsor mandate (a common motivation in academia). Patient groups have become more sophisticated in their approach to shaping the research agenda for their disease conditions. There are positive case studies within rare disease networks that involve patient groups who are phenotypically similar establishing networks, and major stakeholders vested in solutions who are collaborating to bring about effective therapies [13–16]. Across the clinical trials enterprise, there seems to be a natural appreciation of the rationale for involving patient groups earlier in the process. This is in line with other similar patient-centered movements in healthcare, such as the rise of patient-reported outcomes and PCORnet, the National Patient-Centered Clinical Research Network [5]. It is also consistent with increased focus from the U.S. Food and Drug Administration (FDA) [17] as well as the 21^st^ Century Cures Act [18].

Stakeholders need to identify which attributes of patient groups lead to greater partnerships with research sponsors, and all participants in the clinical trial process must embrace the real value of collaboration that should lead to efficiencies and cost savings while producing more relevant outcomes for patients. The goal of our study, therefore, is to provide a snapshot of the different perceptions among stakeholders in the clinical trials enterprise about the importance and value of engaging patient groups. We set out to conduct this foundational work and describe the clinical trial services provided by patient groups as well as potential barriers to successful interactions with industry and academia.

## Participants and Methods

This study was approved by the Duke University School of Medicine Institutional Review Board. Potential participants from patient groups, industry, and academic institutions were identified through rosters and mailing lists of the Clinical Trials Transformation Initiative (CTTI) [19], Drug Information Association, and other stakeholders in the clinical trials enterprise, such as Health Research Alliance and Clinical Research Forum. Individuals were emailed an electronic Qualtrics software (Provo, UT) survey link on May 7, 2014, from CTTI program staff and encouraged to forward the email to their constituencies as a means to increase reach—a method known as snowballing. Snowballing enhances reach but limits the ability to quantify an exact response rate. Survey administration was anonymous, as no identifying information was collected. Two reminder emails were sent during the first 2 weeks of the initial survey mailing.

Survey questions (S1 Appendix Survey) were developed by the authors who represented the three stakeholder groups and informed by a literature review summarizing the available published medical and grey literature (e.g., white papers, government reports) from the past 5 years. Keyword searches and MeSH terms were used, including industry outreach, patient advocacy, clinical trials and research, patient group, and patient involvement. The literature review yielded 22 publications that were referenced in this manuscript. The survey included four domains: (1) importance or value of patient groups in research; (2) clinical trial services provided by patient groups; (3) negative impacts and barriers to relations; and (4) interactions between patient groups and industry and academia. Each domain included several Likert scale, multiple-choice, and “check all that apply” items. The survey also included questions related to the respondent’s affiliation in the patient group, industry, or academic institution.

### Data Analysis Plan

Descriptive statistics were used to examine the characteristics of the organizations represented by the study participants. Chi-square tests were used to compute differences in reporting of frequency of clinical trial services provided by patient groups among the study participants. ANOVA was applied to assess for the differences among study participants in mean scores of the importance or value of patient groups in research. Chi-square tests were used to calculate differences in reporting of frequency of negative impacts to relations between the two pairings: (1) industry and patient group and (2) academia and patient group. Independent t-tests examined the difference between these two pairings in mean scores of satisfaction with relations, engagement priority, and importance in establishing partnerships. A two-sided significance level of 0.05 was used for all statistical tests.

## Results

A total of 179 respondents completed the survey: 24% (n = 43) from industry, 42% (n = 75) from academia, and 34% (n = 61) from patient groups (Table 1). The majority of industry respondents (72%) included those with a primary focus in pharmaceutical development; 67% were from organizations with more than 500 employees, and 58% indicated more than 5 therapies on the market. Industry respondents cited 32 unique job titles that are dedicated primarily to patient engagement activities within their respective companies. Of the survey respondents in academia, 97% were from nonprofit institutions, 80% were from institutions with an NIH Clinical and Translational Science Award, and 71% had initiated contact with a patient group. Of the patient group survey respondents, 49% were affiliated with a group that was established more than 20 years ago, 72% had a single disease focus, 85% cited having a medical or scientific advisory board, 46% reported having an annual budget between $500,000 and $9,999,999, and 13% reported having a budget of more than $100,000,000.

**Table 1:** Characteristics of the Study Sample (n = 179).

Characteristic	N	%	Mean ± SD
**Industry participants (n = 43)**			
Primary focus is pharmaceuticals	31	72	
Over 500 employees	29	67	
>5 medicines or treatments on the market	25	58	
**Academia participants (n = 75)**			
Number of years in current role			12.2 **±** 10.5
Postgraduate degree	42	56	
Nonprofit tax status	71	97	
Has a School of Public Health	33	46	
Has a CTSA center	57	80	
Has initiated contact with a patient group	53	71	
**Patient group participants (n = 61)**			
Achieved tax exempt status >20 years ago	29	49	
<10 full-time or part-time employees	43	72	
<25 volunteers	31	52	
Single disease focus	44	72	
Has a medical or scientific advisory board	51	85	
Reaches >500 patients/caregivers	21	38	
Annual budget >$500,000	27	46	
Patient advocacy organization	38	64	

CTSA, Clinical and Translational Science Award; SD, standard deviation

### Importance or Value of Patient Groups in Research

The perceived importance or value of patient groups in research was rated across research development, study design, study execution, and dissemination of results as shown in Table 2. There were significant differences in the mean scores reported across the three groups; in all cases, patient group respondents reported a greater importance or value in their contributions to research than did academic and industry respondents. The areas of most concordance among industry, academia, and patient group participants were in patient group contributions to improving patient retention (mean scores 4.0/4.1/4.5, respectively; p = .02) and accelerating trial accrual (mean scores 4.1/4.1/4.6, respectively; p = .001). More statistically significant differences were found for all other areas, such as the development of research ideas (mean scores 3.7/3.8/4.7; p < .001) and study protocols (mean scores 3.3/3.2/4.1; p < .001) and interpretation of research results (mean scores 3.0/2.9/3.9; p < .001).

**Table 2:** Importance or Value of Patient Groups in Research^*^.

	Industry (n = 38)		Academia (n = 73)		Patient Group (n = 58)		
Contribution	Mean	SD	Mean	SD	Mean	SD	*P*
**Research Development**							
Developing research ideas	3.7	1.3	3.8	0.9	4.7	0.5	**< .001**
Securing research funding	2.8	1.3	3.6	1.0	4.2	0.9	**< .001**
Developing research proposals	3.1	1.3	3.3	1.0	4.2	0.8	**< .001**
Enhancing proposal’s competitiveness	3.1	1.3	3.8	0.9	4.2	0.8	**< .001**
**Study Design**							
Improving patient retention	4.0	1.0	4.1	1.0	4.5	0.7	**.02**
Designing research protocols	3.3	1.4	3.2	1.1	4.1	1.0	**< .001**
Developing research aims	3.2	1.3	3.5	0.9	4.3	0.9	**< .001**
**Study Execution**							
Accelerating clinical trial accrual	4.1	1.0	4.1	1.0	4.6	0.7	**.001**
Increasing amount of tissues or bio-specimens	3.6	1.1	3.8	1.1	4.6	0.7	**< .001**
Ensuring patient safety in trials	3.4	1.2	3.6	1.1	4.3	0.9	**< .001**
**Dissemination of Results**							
Interpreting research results	3.0	1.4	2.9	1.1	3.9	1.0	**< .001**
Publicizing research findings	3.1	1.4	3.9	1.0	4.5	0.8	**< .001**

SD, standard deviation.

* Likert scale used, range 1–5; higher score indicates greater importance or value.

### Clinical Trial Services Provided by Patient Groups

As reported in Table 3, there was some consistency in the reporting of services provided by patient groups to industry and academia in the conduct of clinical trials across the three participant groups, including a >50% response rate across the two categories “Patient recruitment and retention” and “Educating patients and their families/caregivers about research.” However, patient group respondents cited providing several services at higher rates than industry or academic respondents reported utilizing. Other areas cited by patient group respondents at a higher frequency included participation in clinical trial design (frequencies 9/13/23; p = .02), support during interactions with third-party payers regarding research (frequencies 8/9/22; p = .003), tissue banking (frequencies 1/10/18; p = .001), providing funds for research (frequencies 3/17/30; p < .001), and publicizing and disseminating study results (frequencies 5/27/39; p < .001).

**Table 3:** Clinical Trial Services Provided to Industry and Academia by Patient Groups^*^.

	Industry (n = 43)	Academia (n = 75)	Patient Group (n = 61)	
Clinical trial activity	N (%)	N (%)	N (%)	*P*
**Conduct**				
Patient recruitment and retention	22 (51)	39 (52)	34 (56)	.87
Lack of patient group involvement in the conduct of clinical trials	3 (7)	9 (12)	7 (12)	.67
Providing advice on improving the efficiency of conducting research	4 (9)	4 (5)	6 (10)	.57
Editing informed consent forms	6 (14)	15 (20)	14 (23)	.52
Understanding the trajectory of disease burden	5 (12)	3 (4)	6 (10)	.26
Safety of study participants	5 (12)	19 (25)	13 (21)	.21
Educating patients and their families/caregivers about research	22 (51)	38 (51)	41 (67)	.11
Research report or manuscript development	2 (5)	6 (8)	10 (16)	.11
Interpretation of study results	5 (12)	11 (15)	16 (26)	.10
Bridging with industry	8 (19)	23 (31)	24 (39)	.08
Clinical trial design	9 (21)	13 (17)	23 (38)	**.02**
Support during interactions with third party payers regarding research	8 (19)	9 (12)	22 (36)	**.003**
Tissue banking	1 (2)	10 (13)	18 (30)	**.001**
Funding source for research	3 (7)	17 (23)	30 (49)	**< .001**
Publicity or dissemination of study results	5 (12)	27 (36)	39 (64)	**< .001**
**Dissemination of Results**				
Organized a scientific conference	1 (2)	13 (17)	19 (31)	**.001**
Communicated with the press	1 (2)	14 (19)	20 (33)	**.001**
Website	3 (7)	19 (25)	48 (79)	**< .001**
Newsletter	2 (5)	19 (25)	44 (72)	**< .001**
Presented at a scientific conference	2 (5)	7 (9)	26 (43)	**< .001**
Social media postings	3 (7)	15 (20)	39 (64)	**< .001**

*Check-all-that-apply questions; Chi-square tests performed.

Regarding the dissemination of study results, patient groups cited providing services in greater frequency than industry and academia reported receiving. These services included the organization of scientific conferences (frequencies 1/13/19; p = .001), communication with the press (frequencies 1/14/20; p = .001), dissemination on a website (frequencies 3/19/48; p < .001) or in a newsletter (frequencies 2/19/44; p < .001) or through social media (frequencies 3/15/39; p < .001), and presentation of results at a scientific conference (frequencies 2/7/26; p < .001).

### Negative Impacts to Relations

Table 4 examines the negative impacts or barriers to successful engagement among patient groups, industry, and academia in the conduct of clinical trials. The greatest disagreement between patient group respondents and non–patient group respondents in perceptions of negative impacts had to do with the presence of internal bureaucratic processes, patient group lack of understanding of the benefits of partnering with industry and academia, an unwillingness to share information, a lack of interest in the disease, a lack of understanding by industry and academia of the benefits of partnering with patient groups, and a lack of transparency or openness on the part of the other entity (all p < .05). Additional differences in the perception of barriers between academia and patient group participants were reported in the negotiation of intellectual property and indirect costs (both p < .01). Also, most academic respondents (65%) cited opportunities to gain funding from national programs as an important factor in engaging with patient groups, yet one-third received no patient engagement training and experienced internal resistance or lack of buy-in that impeded their ability to engage with patient groups.

**Table 4:** Negative Impacts to Relations Between Patient Groups and Industry/Academia^*^.

	Industry (n = 43)	Patient Group (n = 61)		Academia (n = 75)	Patient Group (n = 61)	
Negative Impact	N (%)	N (%)	*P*	N (%)	N (%)	*P*
Bureaucratic processes internally	17 (40)	1 (2)	**< .001**	31 (41)	3 (5)	**< .001**
Negotiating intellectual property	6 (13)	13 (21)	.34	6 (8)	20 (33)	**< .001**
Patient group lack of understanding of the benefits of partnering with industry and academia	11 (26)	5 (8)	**.02**	19 (25)	5 (8)	**.009**
An unwillingness to share information	9 (21)	1 (2)	**.001**	9 (12)	0 (0)	**.005**
Indirect costs	4 (9)	3 (5)	.38	18 (24)	30 (49)	**.002**
Lack of interest in the disease	2 (5)	18 (30)	**.002**	7 (9)	14 (23)	**.03**
Unclear or ill-defined process within patient group	12 (28)	22 (36)	.38	24 (32)	26 (43)	.20
Industry or academia lack of understanding of the benefits of partnering with patient groups	12 (28)	34 (56)	**.005**	17 (23)	38 (62)	**< .001**
Their inability to offer meaningful or useful input	6 (14)	9 (15)	.91	10 (13)	9 (15)	.81
Their lack of transparency or openness	6 (14)	29 (48)	**< .001**	14 (19)	28 (46)	**.001**
No factors have negatively impacted interactions	2 (5)	3 (5)	.95	7 (9)	3 (5)	.33

*Check-all-that-apply questions; Chi-square tests performed.

### Perceptions of Intergroup Interactions

Industry and patient group respondents reported moderate satisfaction with their relations and a “medium” priority for engagement (i.e., non-significant p>.05) (Table 5). However, academic respondents cited higher satisfaction with relations than did patient groups (4.1/3.3; p < .001). Patient group respondents reported greater importance in the need for open communications, clear expectations, and detailed contract execution in establishing effective partnerships than did industry and academic respondents (all p < .05). In addition, patient groups reported a greater importance of the need for financial benefit to both parties than did industry respondents (frequencies 2.8/3.7; p = .002).

**Table 5:** Perception of Interactions Between Patient Groups and Industry/Academia.

	Industry (n = 39)	Patient Group (n = 51)		Academia (n = 73)	Patient Group (n = 58)	
Interactions	Mean (SD)	Mean (SD)	*P*	Mean (SD)	Mean (SD)	*P*
Satisfaction with relations*	3.3 (1.0)	3.2 (0.9)	.73	4.1 (0.7)	3.3 (1.0)	**< .001**
Engagement priority (low/med/high)†	2.2 (0.7)	2.3 (0.8)	.81	2.3 (0.7)	2.2 (0.8)	.41
Importance in establishing partnerships‡						
Open communications	4.4 (0.8)	4.8 (0.4)	**.006**	4.6 (0.6)	4.9 (0.3)	**.006**
Clear expectations	4.4 (0.8)	4.9 (0.3)	**.002**	4.6 (0.7)	4.9 (0.3)	**.002**
Detailed contract execution	3.6 (1.3)	4.2 (1.0)	**.02**	3.3 (1.2)	4.1 (1.2)	**< .001**
Financial benefit to both parties	2.8 (1.3)	3.7 (1.2)	**.002**	3.1 (1.4)	3.3 (1.5)	.44
Non-financial benefit to both parties	4.0 (1.0)	4.4 (0.7)	.06	4.3 (0.8)	4.2 (1.0)	.55

SD, standard deviation.

*Likert scale used, range 1–5; higher score indicates greater satisfaction.

†Likert scale used, range 1–3; higher score indicates greater priority.

‡ Likert scale used, range 1–5; higher score indicates greater importance.

## Discussion

This study demonstrates real differences among stakeholder groups in perceptions of the value of patient group engagement with academia and industry around clinical trials—a finding that may represent a significant barrier to engagement that was not identified by the individual stakeholder groups independently. Differences in perceptions may lead to miscommunication and mismatched expectations for these partnerships and should be recognized in the development of tools or guidelines meant to streamline interactions with patient groups. Developing a methodology for assigning a value to the contributions of patient groups in the CTE in absolute terms may also be useful for aligning stakeholders on the issue of valuation.

Most patient group participants reported their ability to provide services in traditional areas such as patient recruitment and retention, patient/family education, and dissemination of study results. Our findings are largely consistent with a recent survey of 201 disease advocacy organizations that reported providing assistance with patient recruitment, data collection, financial support, and study design [20]. However, industry and academic participants reported significantly less receipt of services related to dissemination of study results than that reported by patient groups. A possible explanation is that the research teams were not notified of the publicity efforts; hence, this may be an area of opportunity to enhance industry and academic perceptions of patient group value and contributions and thereby enhance meaningful engagement across the sectors. In addition, our findings related to patient group services reflect the expanding role of patient groups. For example, social media such as Facebook and Twitter are increasingly being used to raise awareness and recruit patients into trials; however, their effectiveness is largely anecdotal [21].

That there is both alignment and difference among stakeholders on perceived barriers to interacting with patient organizations is arguably the most important policy implication to arise from this study. This suggests that, in order to inform the development of best practices, further work is needed to understand which barriers actually have the greatest effect on these relationships. In an emerging field, it is often difficult to know with whom to engage, as demonstrated by the large number of patient engagement job titles reported by our sample. While industry and academia reported moderate rates of internal barriers to engagement, patient groups were more likely to cite external factors, such as a lack of transparency, openness, or understanding of the benefits on the part of industry and academia.

In terms of the importance of establishing partnerships between patient groups, industry, and academia, the high mean scores in open communications and clear expectations reported by the three stakeholder groups reflect the anecdotal evidence. For example, Gallin et al.[2] stress the importance of effective communication, agreement in shared goals, and establishment of appropriate governance structures and processes including oversight of conflicts of interest, scientific rigor, and program evaluation. Another study documented five best practices: (1) vision alignment, (2) resource alignment, (3) partnership structure, (4) management models, and (5) open and frequent communication [22]. It is notable that academic respondents rated their satisfaction with relationships significantly higher than did patient groups. Additional research is needed to understand the factors that contribute to this difference as a means to improve patient group satisfaction.

The strength of our study is in the two-pronged approach to developing the survey questions: (1) questions were informed by a literature review and (2) questions were developed by an author team representing the three stakeholder groups (patient groups, industry, and academia). Study limitations include potential sample bias, as industry respondents may not fully represent a broad spectrum of therapeutic areas, and patient group respondents were largely from more established organizations. In addition, the snowballing method of recruitment may encourage like-minded respondents and may miss clusters of individuals who are not networked with the individuals sampled. Therefore, results may not be generalizable to other, less invested, individuals and groups. In addition, the literature review of patient engagement using MeSH terms revealed little formalized literature and studies to build on. Therefore, we considered it important to employ a three-way stakeholder engagement survey that would reveal more than anecdotal evidence on patient engagement. Last, potential differences may exist between engaging individual patients and organized patient groups.

## Conclusion

Important consistencies and differences exist in perceptions of the value that patient group engagement adds to the clinical trial process. Despite reported similarities between approaches to engagement among industry, academia, and patient groups, key differences exist in perceived barriers and benefits of partnering and engagement that have implications in shaping policy. This recognition could inform the development of best practices. Additional research is needed to define and optimize key success factors for engagement between patient groups, academia, and industry around clinical trials.

## Supporting Information

S1 Appendix Survey (DOCX)

S1 Dataset (DOCX)
