# Supplementary material for: Patient Engagement Practices in Clinical Research among Patient Groups, Industry, and Academia in the United States: A Survey
Source: PLoS One. 2015 Oct 14;10(10):e0140232. doi: 10.1371/journal.pone.0140232 (PMC4605726; doi:10.1371/journal.pone.0140232)
Supplement: S1 Appendix Survey — (DOCX) [file pone.0140232.s001.docx]

WELCOME  Welcome!  This survey focuses on: Understanding interactions between patient groups and academic and industry research sponsors   Thank you for taking time to complete this survey.   The information we learn from this study could aid in the development of best practices in patient engagement.    - The answers you give are confidential and anonymous.  - The survey should take you about 15-20 minutes to complete. If you are unsure of how to answer a question, please give the best answer you can.- Do not exit out of the survey or web browser until you've completed the survey. - You may contact Bray Patrick-Lake via email at bray.patrick-lake@duke.edu or phone at 919-384-5785 if you have any questions, or would like assistance in completing the survey.

AFFILIATIO Please indicate your affiliation.

- Industry (e.g., biopharmaceutical company, biotechnology company, device manufacturer, diagnostics) (1)
- Academic Institution (e.g., university, medical center) (2)
- Patient Group (e.g., patient advocacy, voluntary health, disease advocacy, or public health organization) (3)
- Other (4) ____________________

INTRO-INDU   Please answer the following questions to the best of your ability.     Some questions are specific to your experience, and some questions will ask you what is going on in your company or organization.    Please use the following definitions throughout the survey: Patient groups include patient advocacy, voluntary health, disease advocacy, and public health organizations. Academic institutions include universities, colleges, and medical health centers. Industry includes biopharmaceutical, biotechnology, and diagnostic companies and device manufacturers.

I_PGSTATUS Does your company involve patient advocacy, voluntary health, or public health organizations (i.e., patient groups) in activities that support clinical trials? These are often called "patient engagement" activities.

- No, but my company plans to begin patient engagement activities in the future (2)
- No, and there are no plans at my company to begin patient engagement activities in the future (3)
- Yes (1)
- Don't know (7)

If Yes Is Selected, Then Skip To   The following questions ask about p...If Don't know Is Selected, Then Skip To Congratulations - you have completed ...If Don't know Is Selected, Then Skip To Congratulations - you have completed ...If No, and there are no plans ... Is Selected, Then Skip To Congratulations - you have completed ...

I_NOFUTURE   The following two questions will ask about your company&#39;s plans in conducting patient engagement activities in the future.    Please answer these questions to the best of your ability.     You will then be done with the survey.

I_PGOBJ How important are these objectives to your company in its future patient engagement program?

|  | Extremely (5) | Very (4) | Moderately (3) | A little (2) | Not at all (1) | Don't know (7) |
| --- | --- | --- | --- | --- | --- | --- |
| To learn about patients' unmet needs (1) |  |  |  |  |  |  |
| To promote disease awareness (2) |  |  |  |  |  |  |
| To educate the public, patients, and practitioners about disease (3) |  |  |  |  |  |  |
| To learn about natural disease history and/or the disease experience (4) |  |  |  |  |  |  |
| Other (5) |  |  |  |  |  |  |
| To obtain input on clinically significant endpoints for treatment benefit (6) |  |  |  |  |  |  |
| To obtain input on patients' perspective on risk-benefit balance (8) |  |  |  |  |  |  |
| To obtain input on protocol/clinical trial design (9) |  |  |  |  |  |  |
| To obtain input on clinical operations design (10) |  |  |  |  |  |  |
| To obtain participation in data oversight committee (11) |  |  |  |  |  |  |
| To identify patient population for clinical trial participation (12) |  |  |  |  |  |  |
| To obtain input on development of educational materials (13) |  |  |  |  |  |  |
| To provide knowledge about treatment and treatment options (14) |  |  |  |  |  |  |
| To obtain input on adherence strategies/approaches (15) |  |  |  |  |  |  |
| To promote product awareness (16) |  |  |  |  |  |  |
| To assist with access and reimbursement for products (17) |  |  |  |  |  |  |
| To promote brand awareness (18) |  |  |  |  |  |  |
| To educate the public, patients, and practitioners about clinical trials (20) |  |  |  |  |  |  |
| Don't know (21) |  |  |  |  |  |  |

I_TIMEFRAM What is the approximate time-frame to begin your company's future patient engagement activities?

- Within the next year (1)
- Within the next 1-4 years (2)
- Within 5 or more years (3)
- Don't know (7)

If Within the next year Is Selected, Then Skip To Congratulations - you have completed ...If Within the next 2-4 years Is Selected, Then Skip To Congratulations - you have completed ...If Within 5 or more years Is Selected, Then Skip To Congratulations - you have completed ...If Don't know Is Selected, Then Skip To Congratulations - you have completed ...

I_TEXT   The following questions ask about patient engagement activities at your company.    Please answer these questions to the best of your ability.

I_PHASE At which phase(s) of development does your company engage patients? (Choose all that apply)

- Discovery or pre-clinical phase (1)
- Phase I/Proof of concept (Development decision) (1)
- Phase IIa (1)
- Phase III (1)
- After approval (Launch phase) (1)
- Post-launch (1)
- Don't know (1)
- Other (1) ____________________

I_OBJS How important are these objectives to your company in practicing patient engagement?

|  | Extremely (5) | Very (4) | Moderately (3) | A little (2) | Not at all (1) | Don't know (7) |
| --- | --- | --- | --- | --- | --- | --- |
| To learn about patients' unmet needs (1) |  |  |  |  |  |  |
| To promote disease awareness (2) |  |  |  |  |  |  |
| To educate the public, patients and/or practitioners about disease (3) |  |  |  |  |  |  |
| To learn about natural disease history and/or the disease experience (4) |  |  |  |  |  |  |
| To obtain input on clinically significant endpoints for treatment benefit (5) |  |  |  |  |  |  |
| To obtain input on patients' perspective on risk-benefit balance (6) |  |  |  |  |  |  |
| To obtain input on protocol/clinical trial design (7) |  |  |  |  |  |  |
| To obtain input on clinical operations design (8) |  |  |  |  |  |  |
| To obtain participation in data oversight committee (9) |  |  |  |  |  |  |
| To identify patient population for clinical trial participation (10) |  |  |  |  |  |  |
| To obtain input on development of educational materials (11) |  |  |  |  |  |  |
| To provide knowledge about treatment and treatment options (12) |  |  |  |  |  |  |
| To obtain input on adherence strategies or approaches (13) |  |  |  |  |  |  |
| To promote product awareness (14) |  |  |  |  |  |  |
| To assist with access and reimbursement for products (15) |  |  |  |  |  |  |
| To promote brand awareness (16) |  |  |  |  |  |  |
| To educate the public, patients, and/or practitioners about clinical trials (17) |  |  |  |  |  |  |
| Other (18) |  |  |  |  |  |  |

I_ELECTRON Which electronic methods does your company currently use as a means of engaging patients or patient groups in your company? (Please choose all that apply)

- Use of an informational website (1)
- Use of a company-maintained social media program(s) (1)
- Other (1) ____________________
- Don't know (1)
- Putting company information on a disease-specific organization website (1)
- Through an online patient registry (1)
- Webinars administered by your company (1)
- Email list-serve or distribution list maintained by our company (1)
- We don't use any electronic or digital methods of engaging patients and/or patient groups (1)
- Webinars administered by a patient group(s) (1)
- Email list-serve or distribution list maintained by a patient group(s) (1)
- Use of a general (e.g., Facebook, Twitter, YouTube) social media program(s) (1)

If Use of social media program(s) Is Not Selected, Then Skip To Which non-electronic methods does you...

I_SOCMEDIA What social media programs do you use for engaging with patient groups? (Please choose all that apply)

- Twitter (1)
- Facebook (1)
- YouTube (1)
- Pinterest (1)
- Snapchat (1)
- Instagram (1)
- Tumblr (1)
- Other (1) ____________________
- We do not use social media programs for engaging with patient groups (1)
- Don't know (1)

I_NONELECT Which non-electronic methods does your company currently use as a means of engaging patients or patient groups in your company? (Please choose all that apply)

- Printed educational materials or publications (1)
- Medical call line (1)
- Meetings with patient organizations (1)
- Meetings and/or interviews with individual patients (1)
- Invitations to patients to receive education from company staff (1)
- Invitations to patients to provide education to company staff (1)
- Collaborations on patient-initiated projects (1)
- Provision of grants/funding to patient groups (1)
- Maintain a patient registry (1)
- Involve patient groups in decision-making (1)
- Provision of product access or reimbursement services (1)
- Organization of other patient support services (please specify) (1) ____________________
- Represent patient voice in company decision-making (1)
- Other (1) ____________________
- Don't know (1)
- Meetings and/or interviews with multiple patients (focus groups) (1)
- Network with patient groups at major conferences (1)
- Coalition activities (1)
- We don't use any methods of engaging patients and/or patient groups (1)
- Use of a patient registry maintained by a patient group (1)

I_COLLAB How does your company collaborate with patient groups regarding clinical trials? (Please choose all that apply)

- Provision of patient education (1)
- Involvement in clinical trial design (1)
- Input on clinical trial recruitment and retention (1)
- Advice on regulatory issues (1)
- Insights into commercial pursuits (1)
- Awareness of policy issues (1)
- Other (1) ____________________
- Don't know (1)
- Recruitment through a patient group (1)
- Recruitment through a registry maintained by a patient group (1)

I_CONC1 Describe your level of satisfaction regarding interactions with patient groups.

- Very satisfied (5)
- Satisfied (4)
- Neutral (3)
- Dissatisfied (2)
- Very dissatisfied (1)
- Don't know (7)
- Our company does not interact or partner with patient groups (6)

I_CONC2 What negative factors, if any, has your company experienced when interacting with patient groups? (Please choose all that apply)

- My company's bureaucratic processes (1)
- Negotiating intellectual property (1)
- My company's lack of understanding of the benefits of partnering with patient groups (1)
- My company's unwillingness to share information (1)
- Indirect costs (1)
- Lack of interest in the disease (1)
- Other (1) ____________________
- No factors have negatively impacted our interactions with patient groups (1)
- Don't know (1)
- Unclear or ill-defined process within the patient group(s) (1)
- The patient group(s)' lack of understanding of the benefits of partnering with Industry (1)
- Patient group has not been able to offer meaningful or useful input (1)
- Lack of transparency or openness in communication on the part of patient groups (1)

I_CONC3 What services do patient groups provide in the conduct of clinical trials at your company? (Please choose all that apply)

- Don't know (1)
- Funding source for research (1)
- Tissue banking (1)
- Educating patients and their families/caregivers about research (1)
- Input on informed consent forms (1)
- Patient recruitment and retention (1)
- Understanding the trajectory of disease burden (1)
- Support during interactions with third party payers regarding research (1)
- Bridging with Industry (e.g., pharmaceutical companies, device manufacturers) (1)
- Providing advice on improving the efficiency of conducting research (1)
- Other (1) ____________________
- Clinical trial design (1)
- Interpretation of study results based on clinical meaningfulness (1)
- Safety of design for study participants (1)
- Publicity or dissemination of study results (1)
- Patient groups do not provide support in the conduct of clinical trials at our company (1)
- Research report or manuscript development (1)
- Understanding trial burden (25)
- Exploring the perceived benefits of trial participation (26)
- Providing advice on the development of recruitment and retention materials (27)

If Publicity or external aware... Is Not Selected, Then Skip To What are your objectives when working...

I_CONC4 What avenues have patient groups used to help you support the dissemination of results from clinical trials conducted by your company? (Please choose all that apply)

- Website (1)
- Newsletter (1)
- Patient group organized a scientific conference to present our results (1)
- Communicated with the press (1)
- Other (1) ____________________
- Patient group presented our results at a scientific conference (1)
- Don't know (1)
- Social media postings (1)

I_CTOBJ What are your objectives when engaging with patient groups that are relevant to clinical trials? (Please indicate status of each objective)

|  | Meeting objective now through patient engagement (1) | Planned for future through patient engagement (2) | No plans to meet objective due to constraints (3) | No interest in meeting objective (4) | Don't know (7) |
| --- | --- | --- | --- | --- | --- |
| To learn about patients' unmet needs (2) |  |  |  |  |  |
| To provide knowledge about treatment and treatment options (3) |  |  |  |  |  |
| To promote disease awareness (4) |  |  |  |  |  |
| To educate the public, patients, and practitioners about clinical trial availability (5) |  |  |  |  |  |
| To educate the public, patients, and practitioners about the objectives of clinical product development (6) |  |  |  |  |  |
| To understand the disease experience (8) |  |  |  |  |  |

I_CTDOBJ What are your objectives when engaging with patient groups that are relevant to Clinical Trial Design? (Please indicate status of each objective)

|  | Meeting objective now through patient engagement (1) | Planned for future through patient engagement (2) | No plans to meet objective due to constraints (3) | No interest in meeting objective (4) | Don't know (7) |
| --- | --- | --- | --- | --- | --- |
| To obtain input on protocol/clinical trial design (2) |  |  |  |  |  |
| To obtain input on clinical operations design (3) |  |  |  |  |  |
| To learn about natural disease history (4) |  |  |  |  |  |
| To obtain input on clinically significant endpoints for treatment benefit (5) |  |  |  |  |  |
| To obtain input on patients' perspective on risk-benefit balance (6) |  |  |  |  |  |
| To obtain input on adherence strategies/approaches (7) |  |  |  |  |  |
| To obtain patient-reported outcomes to track quality of life (8) |  |  |  |  |  |
| To learn about the disease experience (10) |  |  |  |  |  |

I_CTROBJ What are your objectives when engaging with patient groups that are relevant to Clinical Trial Recruitment and Retention? (Please indicate status of each objective)

|  | Meeting objective now through patient engagement (1) | Planned for future through patient engagement (2) | No plans to meet objective due to constraints (3) | No interest in meeting objective (4) | Don't know (7) |
| --- | --- | --- | --- | --- | --- |
| To identify patient population for clinical trial participation (2) |  |  |  |  |  |
| To access existing patient registries (3) |  |  |  |  |  |
| To obtain input on written materials for the patient (4) |  |  |  |  |  |
| To obtain information and assistance with ensuring adequate retention (5) |  |  |  |  |  |

I_CTREGOBJ What are your objectives when engaging with patient groups that are relevant to Regulatory Issues? (Please indicate status of each objective)

|  | Meeting objective now through patient engagement (1) | Planned for future through patient engagement (2) | No plans to meet objective due to constraints (3) | No interest in meeting objective (4) | Don't know (7) |
| --- | --- | --- | --- | --- | --- |
| To obtain patient participation on data oversight committee (2) |  |  |  |  |  |
| Fast-tracking/creating alternative pathways to FDA approval (3) |  |  |  |  |  |
| Support for NDA/IDE filings (4) |  |  |  |  |  |
| Patient Preference around Risk/Benefit (5) |  |  |  |  |  |
| Compassionate Use/Continued Access (6) |  |  |  |  |  |
| Expert/Experimental testimonials (Pre-IND, Investigational) (7) |  |  |  |  |  |
| Post-market surveillance (9) |  |  |  |  |  |

I_CTMED What are your objectives when engaging with patient groups that are relevant to Medical? (Please indicate status of each objective)

|  | Meeting objective now through patient engagement (1) | Planned for future through patient engagement (2) | No plans to meet objective due to constraints (3) | No interest in meeting objective (4) | Don't know (7) |
| --- | --- | --- | --- | --- | --- |
| To obtain patient participation on data oversight committee (2) |  |  |  |  |  |
| Post-marketing commitments (Safety/Efficacy) (3) |  |  |  |  |  |
| Emerging standards of care (4) |  |  |  |  |  |
| Adherence/Compliance with current therapies (5) |  |  |  |  |  |
| Tiers of care (e.g., first, second, third line therapies) (6) |  |  |  |  |  |

I_CTCOM What are your objectives when engaging with patient groups that are relevant to Commercial? (Please indicate status of each objective)

|  | Meeting objective now through patient engagement (1) | Planned for future through patient engagement (2) | No plans to meet objective due to constraints (3) | No interest in meeting objective (4) | Don't know (7) |
| --- | --- | --- | --- | --- | --- |
| To promote product awareness (2) |  |  |  |  |  |
| To assist with access and reimbursement for products (3) |  |  |  |  |  |
| To promote brand awareness (4) |  |  |  |  |  |

I_CTPOLICY What are your objectives when engaging with patient groups that are relevant to Policy Issues? (Please indicate status of each objective)

|  | Meeting objective now through patient engagement (1) | Planned for future through patient engagement (2) | No plans to meet objective due to constraints (3) | No interest in meeting objective (4) | Don't know (7) |
| --- | --- | --- | --- | --- | --- |
| Reimbursement - Funding (2) |  |  |  |  |  |
| Legislative Advocacy (FDA, CMS-Payers) (3) |  |  |  |  |  |
| Reform - Health delivery changes to access (4) |  |  |  |  |  |
| Reform - Health Delivery changes to insurance coverage (6) |  |  |  |  |  |
| Reform - Health delivery changes to out-of-pocket deductibles (7) |  |  |  |  |  |

I_CONC5 How much do you agree that patient groups bring value or importance to your company in. . .

|  | Strongly agree (5) | Agree (4) | Neither agree or disagree (3) | Disagree (2) | Strongly disagree (1) | Don't know (7) |
| --- | --- | --- | --- | --- | --- | --- |
| The development of research ideas? (2) |  |  |  |  |  |  |
| Securing research funding? (3) |  |  |  |  |  |  |
| Designing research protocols? (4) |  |  |  |  |  |  |
| Accelerating clinical trial accrual? (5) |  |  |  |  |  |  |
| Increasing the amount of tissues and bio-specimens that are available for research? (6) |  |  |  |  |  |  |
| Ensuring patient safety in clinical trials? (7) |  |  |  |  |  |  |
| Interpreting research results? (8) |  |  |  |  |  |  |
| Publicizing the research findings? (9) |  |  |  |  |  |  |
| Developing research aims? (10) |  |  |  |  |  |  |
| Developing research proposals? (11) |  |  |  |  |  |  |
| Enhancing the proposal's competitiveness? (12) |  |  |  |  |  |  |
| Improving patient retention? (13) |  |  |  |  |  |  |

I_PGVALUE How much do you agree that patient groups bring value or importance to your company in. . .

|  | Strongly agree (5) | Agree (4) | Neither agree or disagree (3) | Disagree (2) | Strongly disagree (1) | Don't know (7) |
| --- | --- | --- | --- | --- | --- | --- |
| Enhancing awareness and understanding of the burden/impact of disease/condition on the patient population? (3) |  |  |  |  |  |  |
| Publicizing/disseminating information on the progress of ongoing research to patient communities? (4) |  |  |  |  |  |  |
| Disseminating knowledge about therapeutic and treatment options? (10) |  |  |  |  |  |  |
| Improving patient access to therapies and treatments? (20) |  |  |  |  |  |  |

I_CONC6 How important are these factors in establishing partnerships with patient groups as they relate to clinical trials?

|  | Extremely (5) | Very (4) | Moderately (3) | A little (2) | Not at all (1) | Don't know (7) |
| --- | --- | --- | --- | --- | --- | --- |
| Open communications and transparency (2) |  |  |  |  |  |  |
| Clear expectations (3) |  |  |  |  |  |  |
| Execution of detailed contracts (4) |  |  |  |  |  |  |
| Financial benefit to both parties (your company and the organization) (5) |  |  |  |  |  |  |
| Benefits (other than financial) to both parties (6) |  |  |  |  |  |  |

I_STRATEGY What drives your company's strategic thinking in developing patient engagement objectives? (Please choose all that apply)

- Research considerations (1)
- Regulatory considerations (1)
- Reimbursement considerations (1)
- Other (1) ____________________
- Don't know (1)
- Public relations (1)

I_FACTORS What internal factors at your company drive you to engage with patient groups? (Please choose all that apply)

- Early - Proof of concept (1)
- Phased clinical trial support (1)
- Therapeutic Area Alignment dependent (1)
- Mature Patient Engagement/Outreach in established departments (1)
- Other (1) ____________________
- Don't know (1)

I_APPROACH What is your company's most important consideration in its approach to working with patient groups?

- Vertical business unit dependent - Therapeutic area focus (1)
- Company culture (2)
- Funding/Market opportunities - Pipeline dependent for post-marketing (3)
- Investigator initiated vs. Corporate focus (4)
- Other (5) ____________________
- Don't know (7)

I_CONC7 Is engaging with patient groups to support the conduct of clinical trials a high, medium or low priority within your company?

- High (3)
- Medium (2)
- Low (1)
- Don't know (7)

I_DETERMIN How does your company determine which patient groups to engage with? (Please choose all that apply)

- Pipeline priorities (1)
- History of leadership in a therapeutic area (1)
- Assessment of patient group's capabilities (national vs. regional focus, community reach, patient network, KOLS network, research programs, advocacy programs) (1)
- Legal considerations (1)
- Other (1) ____________________
- Don't know (1)

I_CHAR What characteristics are most important to your company in identifying patient groups to engage with? (Please choose all that apply)

- We don't consider patient group characteristics (1)
- Disease focus (1)
- National vs. regional reach (1)
- Community reach (1)
- Patient network/patient education programs (1)
- Access to tissue/samples (1)
- Other (1) ____________________
- KOLs network (1)
- Research programs (1)
- Advocacy programs (1)
- Don't know (1)
- Organizational structure (1)
- Leadership expertise and capacity (1)

I_R&D At what point in the research and development process does your company engage with patient groups? (Please choose all that apply)

- Early Pipeline Planning (1)
- Lead and Target Identification (1)
- Development of specific R&D Plans around a molecule (1)
- Launch of Phase 1, 2, or 3 Clinical Trials (1)
- Around FDA Filing (1)
- Post Market (1)
- Other (1) ____________________
- Don't know (1)

I_RELEVANT What areas are most relevant to your company in engaging with patient groups? (Please choose all that apply)

- Research & Development (1)
- Regulatory (1)
- Patient Safety/Communication Benefit vs. Risk (1)
- Reimbursement (1)
- Other (1) ____________________
- Don't know (1)

I_METRICS How are you measuring the impact of your company&#39;s patient engagement activities? (Please choose all that apply)

- We are not measuring the impact (1)
- Trial accrual rates (1)
- Retention, % subjects retained (1)
- Cycle time metrics in general (1)
- Minimal protocol amendments (1)
- Limiting unnecessary cost outlays (1)
- Other (1) ____________________
- Don't know (1)

I_CONTACT Who are your main sources of contact within patient groups? (Please choose all that apply)

- We don't have any main sources of contact within patient groups (1)
- CEO/Executive Director (1)
- Research /Medical Program leads (1)
- Development staff (1)
- Patient/caregiver volunteers (1)
- Other (1) ____________________
- Don't know (1)

I_BARRIERS What barriers exist in your ability to fully execute your patient engagement program? (Please choose all that apply)

- We don't have any barriers in our ability to fully execute our patient engagement program (1)
- Lack of internal company buy-in (1)
- Misaligned objectives between my company and patient groups (1)
- Lack of funding (1)
- Legal constraints (1)
- Unsure of which patient groups to engage with (1)
- Unsure of how to best engage with patient groups (1)
- Other (1) ____________________
- Lack of sophistication among patient groups (1)
- Insufficient tools for identifying/engaging with relevant patient groups (1)
- Don't know (1)

I_POSITION How many positions in your company are dedicated mainly to patient engagement activities?

- None, but patient engagement activities are integrated into several positions in my company (5)
- One (1)
- Two (2)
- Three (3)
- Don't know (7)
- None, and patient engagement activities are NOT integrated into other positions (6)
- More than three (4)

If None, and patient engagemen... Is Selected, Then Skip To Please describe your company's most s...If None, and patient engagemen... Is Selected, Then Skip To Please describe your company's most s...If Don't know Is Selected, Then Skip To Please describe your company's most s...

I_POSTITLE Please specify the title(s) and reporting function(s) for the positions in your company that are dedicated mainly to patient engagement activities.

I_SUCCESS Please describe your company&#39;s most successful patient engagement effort.

I_FOCUS What is the primary focus of your company? (Please choose all that apply)

- Pharmaceuticals (1)
- Biologics (1)
- Generics (1)
- Devices, including combination products (1)
- Contract service organization (1)
- Other (1) ____________________
- Don't know (1)

I_NUMEMPL How many paid (full and part time) employees work for your company?

- 10 - 500 employees (small business) (2)
- Less than 10 (small office) (1)
- Over 500 employees (3)
- Don't know (4)

I_NUMMARKE How many medicines or treatment products does your company have on the market?

- 0 (1)
- 1 (2)
- Don't know (7)
- More than 5 (4)
- 2 - 5 (3)

I_NUMDEVT How many medicines or treatment products does your company have in development?

- 0 (1)
- 1 (2)
- Don't know (7)
- More than 5 (4)
- 2 - 5 (3)

I_CODISEAS What are the primary disease or therapeutic focus areas of your company? (Please choose all that apply)

- Cardiovascular (1)
- Don't know (1)
- Diabetes (1)
- CNS (1)
- Oncology (1)
- Rare diseases (1)
- Other (1) ____________________

I_PGDISEAS What disease or therapeutic focus areas are included in your patient engagement or advocacy efforts? (Please choose all that apply)

- Cardiovascular (1)
- Don't know (1)
- Diabetes (1)
- CNS (1)
- Oncology (1)
- Rare diseases (1)
- Other (1) ____________________

I_FUNDS How frequently does your company allocate funds to patient engagement or advocacy organization efforts?

- Annual budget for patient group projects/initiatives (1)
- Don't know (7)
- Set amount given to specific patient groups each year (2)
- No funds provided (4)
- Decisions made project by project based on alignment with department/company objectives (3)

If Our company does not alloca... Is Selected, Then Skip To Is your company interested in partici...If Don't know Is Selected, Then Skip To Is your company interested in partici...

I_EFFORTS What types of efforts does your company fund? (Please choose all that apply)

- Open grant to organization (1)
- Company-specified initiatives (please specify below) (1) ____________________
- Patient organization proposals (please specify below) (1) ____________________
- Other (1) ____________________
- Don't know (1)

I_INTEREST Is your company interested in participating in collaborative patient engagement or advocacy events in 2014?

- Yes (1)
- No (0)
- Don't know (7)

If Yes (Please specify topic(s... Is Not Selected, Then Skip To Congratulations - you have completed ...

I_TOPICS Please specify collaborative patient engagement or advocacy event topics of interest in 2014.

I_EMAIL Please provide your email address to receive notification for collaborative patient engagement or advocacy event in 2014.

I_ENDOFSUR Congratulations - you have completed the survey!   Your answers are extremely important to us and your efforts are greatly appreciated.   PLEASE NOTE! None of your answers will be recorded until you click the 'NEXT' button below.  If you would like to go back at this time to change any answers, you may do so now.  Otherwise, to record your final answers, you must click the NEXT button.

INTRO-ACA   Please answer the following questions from the point of view of being a research professional at your academic institution (e.g., university, medical center).      Some questions are specific to your experience with patient groups (NOTE: patient representatives are NOT included in this definition), and some questions will ask you what is going on in your institution.     Please use the following definitions throughout the survey: Patient groups include patient advocacy, voluntary health, disease advocacy, and public health organizations. Academic institutions include universities, colleges, and medical health centers. Industry includes biopharmaceutical, biotechnology, and diagnostic companies and device manufacturers.

A_ROLE What is your role(s) at your academic institution? (Please choose all that apply)

- Clinical research investigator (1)
- Basic science investigator (1)
- Member of Institutional Review Board (1)
- Other investigator (1) ____________________
- Administrator (1)
- Educator (1)
- Other (1) ____________________
- Outcomes research investigator (1)
- Clinician (1)

A_YEARSEXP How many years have you been in your current role?

______ Number of years (1)

A_DEGREE What is your highest educational degree?

- High school or technical school (1)
- Bachelor's degree (e.g., BA, BS) (3)
- Master's level or equivalent (e.g., MA, MS, MBA) (4)
- Post-graduate degree (e.g., PhD, MD) (5)
- Other (6) ____________________
- Some college (2)

A_TAXSTAT What is the tax status of your academic institution?

- For Profit (1)
- Not for Profit (2)
- Don't know (7)

A_PUBHLTH Does your academic institution have a School of Public Health?

- No (0)
- Yes (1)
- Dont' know (7)

A_CTSA Does your institution have a Clinical Translational Science Award (CTSA) center or related institute? (To learn more about what a CTSA is, go to http://www.ncats.nih.gov/research/cts/ctsa/about/about.html)

- No (0)
- Yes (1)
- Dont' know (7)

A_TRAINING What type of training and education have you received to engage or interact with patient groups? (Please choose all that apply; A patient group is defined as a patient advocacy, voluntary health, or public health organization)

- I received no training or education on engaging or interacting with patient groups (1)
- Informal training (e.g., blogs, website reviews) (1)
- Institutional training module (1)
- Other (1) ____________________
- One-on-one advice and training from a colleague who has done this (1)
- One-on-one advice and training from a patient representative who has done this (1)

A_ENGAGE Please describe your engagement or interaction with patient groups. (Please choose all that apply)

- I never engaged with patient groups (1)
- I have initiated contact with patient groups (1)
- Patient groups have contacted me for engagement (1)
- Other (1) ____________________

If I never engaged with patien... Is Selected, Then Skip To Is engaging with patient groups to su...

A_CONC1 Please describe your level of satisfaction regarding interactions with patient groups.

- Very satisfied (5)
- Satisfied (4)
- Neutral (3)
- Dissatisfied (2)
- Very dissatisfied (1)
- Don't know (7)

A_CONC7 Is engaging with patient groups to support the conduct of clinical trials a high, medium or low priority within your academic institution?

- High (3)
- Medium (2)
- Low (1)
- Don't know (7)

A_CONC6 How important are these factors in establishing partnerships with patient groups as they relate to clinical trials?

|  | Extremely (5) | Very (4) | Moderately (3) | A little (2) | Not at all (1) | Don't know (7) |
| --- | --- | --- | --- | --- | --- | --- |
| Open communications and transparency (2) |  |  |  |  |  |  |
| Clear expectations (3) |  |  |  |  |  |  |
| Execution of detailed contracts (4) |  |  |  |  |  |  |
| Financial benefit to both parties (patient group and the institution) (5) |  |  |  |  |  |  |
| Benefits (other than financial) to both parties (6) |  |  |  |  |  |  |

A_CONC2 What negative factors, if any, has your institution experienced when interacting with patient groups? (Please choose all that apply)

- My institution's bureaucratic processes (1)
- Negotiating intellectual property (1)
- My institution's lack of understanding of the benefits of partnering with patient groups (1)
- My institution's unwillingness to share information (1)
- Indirect costs (1)
- Lack of interest in the disease (1)
- Other (1) ____________________
- No factors have negatively impacted our interactions with patient groups (1)
- Don't know (1)
- Unclear or ill-defined process within the patient group(s) (1)
- The patient group(s)' lack of understanding of the benefits of partnering with Academia (1)
- Patient group has not been able to offer meaningful or useful input (1)
- Lack of transparency or openness in communication on the part of patient groups (1)

A_CONC3 What services do patient groups provide in the conduct of clinical trials at your academic institution? (Please choose all that apply)

- Don't know (1)
- Funding source for research (1)
- Tissue banking (1)
- Educating patients and their families/caregivers about research (1)
- Input on editing informed consent forms (1)
- Patient recruitment and retention (1)
- Understanding the trajectory of disease burden (1)
- Support during interactions with third party payers regarding research (1)
- Bridging with industry (e.g., pharmaceutical companies, device manufacturers) (1)
- Providing advice on improving the efficiency of conducting research (1)
- Other (1) ____________________
- Clinical trial design (1)
- Interpretation of study results based on clinical meaningfulness (1)
- Safety of design for study participants (1)
- Publicity or dissemination of study results (1)
- Patient groups do not provide support in the conduct of clinical trials in our institution (1)
- Research report or manuscript development (1)

If Publicity or external aware... Is Not Selected, Then Skip To In your opinion, what are the reasons...

A_CONC4 What avenues have patient groups used to help you support the dissemination of clinical trial results? (Please choose all that apply)

- Website (1)
- Newsletter (1)
- Patient group organized a scientific conference to present our results (1)
- Communicated with the press (1)
- Other (1) ____________________
- Patient group presented our results at a scientific conference (1)
- Don't know (1)
- Social media postings (1)

A_REASONS In your opinion, what are the reasons that your academic institution has involved patient groups in research activities? (Please choose all that apply)

- We do not have a focus when finding effective collaborations with patient groups (1)
- Mutual respect for potential contributions to the intended activities by each party (1)
- Sharing governance or oversight of specific clinical research projects (1)
- Governance or oversight of institutional research priorities (1)
- Opportunities to gain funding from national programs (e.g., Patient Centered Outcomes Research Institute) (1)
- Opportunities to be funded by patient groups (1)
- Other (1) ____________________
- Don't know (1)

A_MEANINGF How would your institution define "Meaningful Involvement" in building clinical research infrastructure in partnership with patient groups? (Please choose all that apply)

- CTSA-Mandated (1)
- Community Engagement (1)
- NIH Rare Disease alignment - incentives (1)
- NCI mandates or incentives (1)
- Patient and/or family advisory boards (1)
- Patient and/or family education - dissemination (1)
- Other (1) ____________________
- Don't know (1)

A_BARRIERS What barriers exist in your ability to fully engage patient groups? (Please choose all that apply)

- There are no barriers in our ability to fully execute our patient engagement program (1)
- Internal resistance and/or lack of internal academic buy-in (1)
- Other (1) ____________________
- Misaligned objectives, priorities, or incentives between the investigators and patient groups (1)
- Lack of sophistication among patient groups (1)
- Insufficient tools for identifying relevant patient groups (1)
- Lack of sufficient funding (1)
- Legal constraints (1)
- Insufficient tools for engaging with relevant patient groups (1)
- Don't know (1)

A_CONC5 How much do you agree that patient groups bring value or importance to your academic institution in. . .

|  | Strongly agree (5) | Agree (4) | Neither agree or disagree (3) | Disagree (2) | Strongly disagree (1) | Don't know (7) |
| --- | --- | --- | --- | --- | --- | --- |
| The development of research ideas? (2) |  |  |  |  |  |  |
| Securing research funding? (3) |  |  |  |  |  |  |
| Designing research protocols? (4) |  |  |  |  |  |  |
| Accelerating clinical trial accrual? (5) |  |  |  |  |  |  |
| Increasing the amount of tissues and bio-specimens that are available for research? (6) |  |  |  |  |  |  |
| Ensuring patient safety in clinical trials? (7) |  |  |  |  |  |  |
| Interpreting research results? (8) |  |  |  |  |  |  |
| Publicizing the research findings? (9) |  |  |  |  |  |  |
| Developing research aims? (10) |  |  |  |  |  |  |
| Developing research proposals? (11) |  |  |  |  |  |  |
| Enhancing the proposal's competitiveness? (12) |  |  |  |  |  |  |
| Improving patient retention? (13) |  |  |  |  |  |  |

A_COMPLETE Congratulations - you have completed the survey!   Your answers are extremely important to us and your efforts are greatly appreciated.   PLEASE NOTE! None of your answers will be recorded until you click the 'NEXT' button below.  If you would like to go back at this time to change any answers, you may do so now.  Otherwise, to record your final answers, you must click the NEXT button.

PG_INTRO   Please answer the following questions to the best of your ability.     Some questions will ask about your experiences as a decision maker for your patient group, and some will ask you about organizational characteristics and activities.     Please use the following definitions throughout the survey: Patient groups include patient advocacy, voluntary health, disease advocacy, and public health organizations. Academic institutions include universities, colleges, and medical health centers. Industry includes biopharmaceutical, biotechnology, and diagnostic companies and device manufacturers.

PG_TAXEX Is your organization tax-exempt (e.g., non-profit, 501c3, online community)?

- Yes (1)
- No (0)
- Don't know (7)

If Yes Is Selected, Then Skip To What type of patient group are you a ...

PG_INELNP You must represent a non-profit patient group to be eligible for this study.Thank you for your interest!

If You must be a member of a t... Is Displayed, Then Skip To Congratulations - you have completed ...

PG_ORGTYPE Your group refers to itself as a. . .

- Patient advocacy organization: Typically a not-for profit organization that is often founded by families of patients to raise funds from the public (1)
- Voluntary health organization: Typically a not-for-profit organization that derives its revenue primarily from voluntary contributions from the general public to be used for general or specific purposes connected with health, welfare, or community services (2)
- Public health organization or affiliate: These organizations work to prevent disease, promote health, and prolong life with focus on the population as a whole rather than individual patients or diseases (3)
- Other (5) ____________________
- I am not a member of a patient group (4)

If I am not a member of a pati... Is Not Selected, Then Skip To What is your organization's age in ye...

PG_INELPG You must be a member of a patient group to be eligible for this study.   Thank you for your interest!

If You must be a member of a p... Is Displayed, Then Skip To Congratulations - you have completed ...

PG_TAXEXYR When did your organization achieve tax exempt status?

- Less than 5 years ago (1)
- 5-10 years ago (2)
- 11-20 years ago (3)
- Don't know (7)
- More than 20 years ago (4)

PG_NUMEMPL How many paid (full-time and part-time) employees are in your organization?

- Less than 25 employees (1)
- 25-49 employees (2)
- 50-74 employees (3)
- 75-100 employees (4)
- More than 100 employees (5)
- Don't know (7)

PG_NUMVOLS About how many volunteers help staff your organizational operations?

- Less than 25 volunteers (1)
- 25-49 volunteers (2)
- 50-74 volunteers (3)
- 75-100 volunteers (4)
- Don't know (7)
- More than 100 volunteers (5)

PG_DISEASE What is your organization&#39;s disease focus?

- Single disease (please name it here with number afflicted) (1) ____________________
- Multiple diseases (please name them here with number afflicted, separated by commas) (2) ____________________
- Other (3) ____________________

PG_AFFLICT Approximately how many patients are afflicted with this disease(s) in the US?

PG_ADVBD Does your organization have a medical or scientific advisory board?

- No (0)
- Yes (1)
- Don't know (7)

PG_ADVISOR Does your organization have a medical or scientific advisor?

- No (0)
- Yes (1)
- Don't know (7)

PG_REACH Approximately how many patients/caregivers does your organization reach? (Enter number below)

- Less than 1000 (1)
- 1001-5000 (2)
- 5001-10,000 (3)
- 10,001-50,000 (4)
- More than 50,000 (5)
- Don't know (7)

PG_BUDGET What was your annual budget for tax-year 2013?

- Less than $500,000 (1)
- $10,000,000 - $49,999,999 (3)
- $50,000,000 - $99,999,999 (4)
- $100,000,000 or Greater (5)
- Don't know (7)
- $500,000 - $9,999,999 (2)

PG_PERCRES What percentage of your organization's budget is dedicated to supporting research activities?

- 0% (1)
- 1-25% (2)
- 26-50% (3)
- More than 75% (5)
- Don't know (7)
- 51-75% (4)

PG_SERVICE What are the main services or activities of your organization? (Please choose all that apply)

- Building the patient community (locating fellow patients) (1)
- Development and maintenance of patient registry (1)
- Raising awareness of the disease or condition (1)
- Providing education about the disease or condition (1)
- Identifying treatment or care networks (1)
- Providing supportive services for patients (please specify) (1) ____________________
- Providing supportive services for family or caregivers (please specify) (1) ____________________
- Raising funds (1)
- Providing grants or funding to patients for trial participation (1)
- Providing grants or funding to researchers (1)
- Advocating for treatment research and development (1)
- Engaging with companies that are developing treatments (1)
- Advocating for legislation (1)
- Providing product access or reimbursement services (1)
- Other (1) ____________________
- Providing education about specific clinical research studies (1)
- Providing education about participating in clinical trials (1)

PG_DECISIO How are decisions concerning research activities made within your organization? (Please choose all that apply)

- My organization's own review committees (e.g., Medical Science Research Committee, Steering Committee, Research Executive Committee, Lay Review Committee, Grant Review Committee) (1)
- Other (1) ____________________
- Don't know (1)
- Program staff (e.g., Scientific Program Officer, Scientific Program Director, Scientific Program Manager) (1)
- Executive leadership (e.g., CEO/President, Chief Scientific Officer, Chief Medical Officer, SVP/VP/AVP Research, VP/Director research and Scientific Affairs) (1)
- External review committee (some other organization's committee) (1)

If My organization's own revie... Is Not Selected, Then Skip To How are patients/caregivers engaged i...

PG_STAKEHO Does your review committee include patient stakeholders?

- Yes (1)
- No (0)
- Don't know (7)

PG_RESENG How are patients/caregivers engaged in research through your organization? (Please choose all that apply)

- They are on a board that reviews research, such as a scientific advisory board (1)
- They act as grant reviewers (1)
- They participate in a research advocacy training program (1)
- They may choose to be research participants (1)
- Other (1) ____________________
- Patients/caregivers are not engaged in research through my organization (1)
- Don't know (1)

PG_INTRO3Q   The next three questions will cover aspects involving clinical trials:   1. Activities to support clinical trial awareness 2. Services in support of clinical trial planning 3. Services in support of the conduct of clinical trials

PG_CTA What activities are conducted by your organization in support of clinical trials awareness? (Please choose all that apply)

- General patient education documentation and materials about participating in clinical trials (1)
- Newsletter content related to clinical trials (1)
- Support group(s) that focus on clinical trial topics (1)
- Toll-free support line where clinical trials are discussed (1)
- Informational website developed by your organization with clinical trial content (1)
- Links to clinical trial resources on your website (1)
- Public policy maker education and/or lobbying that is relevant to clinical trials (1)
- Financial assistance to patients to participate in clinical trials (1)
- Other (1) ____________________
- Our organization does not conduct any activities in support of clinical trials (1)
- Don't know (1)
- Patient education materials about specific clinical trials (1)
- Links to clinical trial resources on external websites (e.g., ClinicalTrials.gov) (1)

PG_CTP What services are provided by your organization in support of clinical trial planning? (Please choose all that apply)

- Financial support for investigators (1)
- Advice in the design of a research question, protocol, or project (1)
- Letter of support for investigator (1)
- Writing of grant proposal (1)
- Other (1) ____________________
- Our organization does not provide any services in support of clinical trial planning (1)
- Financial support targeted to young investigators (1)
- Don't know (1)
- Provision of feasibility data (e.g., numbers of potential participants in a geographic area) (1)
- Advice on clinical sites/clinician investigators (1)
- Advice on endpoints (1)

PG_CONC3 What services are provided by your organization in support of the conduct of clinical trials? (Please choose all that apply)

- Don't know (1)
- Funding source for research (1)
- Tissue banking (1)
- Educating patients and their families/caregivers about specific research studies (1)
- Reviewing and/or editing consent forms (1)
- Patient recruitment and retention (1)
- Help researchers understand the trajectory of disease burden (1)
- Support during interactions with third party payers regarding research (1)
- Act as a liaison between trial participants and industry for clinical trial issues (1)
- Providing advice on improving the efficiency of conducting research (1)
- Other (1) ____________________
- Interpretation of study results based on clinical meaningfulness (1)
- Safety of design for study participants (1)
- Publicity or dissemination of study results (1)
- Our organization does not provide support in the conduct of clinical trials (1)
- Research report or manuscript development (1)
- Help researcher understand the trial burden (1)
- Explore the perceived benefits of trial participation (1)
- Providing advice on the development of recruitment and retention materials (1)
- Clinical trial design (1)

PG_CTSOINT Do you interact with clinical trial services organizations (CRO, central labs, recruitment services)?

- Yes (1)
- No (0)
- Don't know (7)

If Yes Is Not Selected, Then Skip To What avenues have been used by your o...

PG_CTSOCHA What are your primary challenges in working with clinical trial services organizations? (Choose all that apply)

- Patient travel reimbursement (1)
- Patient travel scheduling (1)
- Scheduling of procedures/labs (1)
- Logistics obtaining procedure/lab results (1)
- Flexibility around study logistics (e.g., shipping blood vs. patient traveling to site) (1)
- Other (1) ____________________

PG_CTSOSTR What are your strategies for resolving study execution issues managed by clinical trial services organizations? (Please choose all that apply)

- Direct communication with clinical trial services organizations (1)
- Having a working relationship with clinical trial services organizations (1)
- Education of research sponsor clinical trial services organization on patient experiences and needs (1)
- Advise participants on how to advocate for themselves to clinical trial services organizations (1)
- Other (1) ____________________

PG_CONC4 What avenues have been used by your organization to support the dissemination of clinical trial results? (Please choose all that apply)

- Website (1)
- Newsletter (1)
- Organized a scientific conference (1)
- Communicated with the press (1)
- Other (1) ____________________
- Presented at a scientific conference (1)
- Don't know (1)
- Social media postings (1)

PG_CONC5 How much do you agree that patient groups bring value or importance to Industry and academic institutions in. . .

|  | Strongly agree (5) | Agree (4) | Neither agree or disagree (3) | Disagree (2) | Strongly disagree (1) | Don't know (7) |
| --- | --- | --- | --- | --- | --- | --- |
| The development of research ideas? (2) |  |  |  |  |  |  |
| Securing research funding? (3) |  |  |  |  |  |  |
| Designing research protocols? (4) |  |  |  |  |  |  |
| Accelerating clinical trial accrual? (5) |  |  |  |  |  |  |
| Increasing the amount of tissues and bio-specimens that are available for research? (6) |  |  |  |  |  |  |
| Ensuring patient safety in clinical trials? (7) |  |  |  |  |  |  |
| Interpreting research results? (8) |  |  |  |  |  |  |
| Publicizing the research findings? (9) |  |  |  |  |  |  |
| Developing research aims? (10) |  |  |  |  |  |  |
| Developing research proposals? (11) |  |  |  |  |  |  |
| Enhancing the proposal's competitiveness? (12) |  |  |  |  |  |  |
| Improving patient retention? (13) |  |  |  |  |  |  |

PG_A_CONC1 Describe your level of satisfaction regarding interactions with academic institutions.

- Very satisfied (5)
- Our organization does not interact or partner with academic institutions (6)
- Satisfied (4)
- Neutral (3)
- Dissatisfied (2)
- Very dissatisfied (1)
- Don't know (7)

If Our organization does not i... Is Selected, Then Skip To How would you describe your interacti...

PG_A_CONC7 Is engaging or partnering with academic institutions (e.g., universities, health centers) to support the conduct of clinical trials a high, medium or low priority within your organization?

- High (3)
- Medium (2)
- Low (1)
- Don't know (7)

PG_A_CONC6 How important are these factors in establishing partnerships with academic institutions as they relate to clinical trials?

|  | Extremely (5) | Very (4) | Moderately (3) | A little (2) | Not at all (1) | Don't know (7) |
| --- | --- | --- | --- | --- | --- | --- |
| Open communications and transparency (2) |  |  |  |  |  |  |
| Clear expectations (3) |  |  |  |  |  |  |
| Execution of detailed contracts (4) |  |  |  |  |  |  |
| Financial benefit to both parties (your organization and the institution) (5) |  |  |  |  |  |  |
| Benefits (other than financial) to both parties (6) |  |  |  |  |  |  |

PG_ACANEED How well do you believe that investigators within academic institutions understand the needs of patients?

- Extremely (5)
- Very (4)
- Moderately (3)
- A little (2)
- Not at all (1)
- Don't know (7)

PG_ACABENE What benefits has your organization received from partnering with academic institutions? (Please choose all that apply)

- Increased our scientific expertise (1)
- Led to discovery of new technologies (1)
- Improved the institution's commitment to discovery (1)
- Enhanced our research & development expertise (1)
- Our support has equipped laboratories (1)
- Other (1) ____________________
- Gained insights into the academic sector (1)
- Gained financial support for our organization (1)
- Don't know (1)
- Improved our own research infrastructure (1)

PG_A_CONC2 What negative factors, if any, has your organization experienced when interacting with academic institutions? (Please choose all that apply)

- My organization's bureaucratic processes (1)
- Negotiating intellectual property (1)
- My organization's lack of understanding of the benefits of partnering with academic institutions (1)
- My organization's unwillingness to share information (1)
- Indirect costs (1)
- Lack of interest in the disease (1)
- Other (1) ____________________
- No factors have negatively impacted our interactions with academic institutions (1)
- Don't know (1)
- Unclear or ill-defined process within the academic institution (1)
- The academic institution(s)' lack of understanding of the benefits of partnering with patient groups (1)
- Academic institution(s) has not been able to offer meaningful or useful input (1)
- Institutional Review Board delays (1)
- Lack of transparency or openness in communication on the part of academic institutions (1)

PG_ACABARR In your opinion, what is the most important barrier to engaging with academic institutions as it relates to clinical trials?

- Unsure of whom to contact in the institution (1)
- Lack of clarity in roles (2)
- Mistrust in regards to the institution's motives (8)
- Institution's reluctance to share data (9)
- Intellectual property issues (10)
- Other (11) ____________________
- Don't know (7)
- Lack of clarity in expectations or anticipated outcomes (6)
- Unsure what value our organization brings to academic researchers (3)
- Academic institutions are not interested in engaging with us (4)
- Academic institutions do not listen to our perspectives (5)

PG_I_CONC1 Describe your level of satisfaction regarding interactions with Industry.

- Very satisfied (5)
- Our organization does not interact or partner with Industry (6)
- Satisfied (4)
- Neutral (3)
- Dissatisfied (2)
- Very dissatisfied (1)
- Don't know (7)

If Our organization does not i... Is Selected, Then Skip To Congratulations - you have completed ...

PG_I_CONC7 Is engaging or partnering with Industry (e.g., pharmaceutical company, device manufacturer) to support the conduct of clinical trials a high, medium or low priority within your organization?

- High (3)
- Medium (2)
- Low (1)
- Don't know (7)

PG_ATTY Have you had to retain or employ an attorney about any of these industry-related issues? (Please choose all that apply)

- Ethical issues (1)
- Intellectual property issues (1)
- Regulatory issues (1)
- Data privacy or data sharing issues (1)
- Other (1) ____________________
- Don't know (1)

PG_INDBENE What benefits has your organization received from partnering with Industry? (Please choose all that apply)

- Increased our commercialization expertise (1)
- Led to discovery of new technologies (1)
- Improved the company's commitment to discovery (1)
- Enhanced our research & development expertise (1)
- Our support has equipped laboratories (1)
- Other (1) ____________________
- Gained insights into the private sector (1)
- Gained financial support for our organization (1)
- Don't know (1)
- Improved our own research infrastructure (1)

PG_I_CONC6 In your opinion, how important are these factors in establishing partnerships with Industry as they relate to clinical trials?

|  | Extremely (5) | Very (4) | Moderately (3) | A little (2) | Not at all (1) | Don't know (7) |
| --- | --- | --- | --- | --- | --- | --- |
| Open communications and transparency (2) |  |  |  |  |  |  |
| Clear expectations (3) |  |  |  |  |  |  |
| Execution of detailed contracts (4) |  |  |  |  |  |  |
| Financial benefit to both parties (your organization and the company) (5) |  |  |  |  |  |  |
| Benefits (other than financial) to both parties (6) |  |  |  |  |  |  |

PG_INDNEED How well do you believe that investigators within the companies that you work with understand the needs of patients?

- Extremely (5)
- Very (4)
- Moderately (3)
- A little (2)
- Not at all (1)
- Don't know (7)

PG_I_CONC2 What negative factors, if any, has your organization experienced when interacting with Industry? (Please choose all that apply)

- My organization's bureaucratic processes (1)
- Negotiating intellectual property (1)
- My organization's lack of understanding of the benefits of partnering with Industry (1)
- My organization's unwillingness to share information (1)
- Indirect costs (1)
- Lack of interest in the disease (1)
- Other (1) ____________________
- No factors have negatively impacted our interactions with Industry (1)
- Don't know (1)
- Unclear or ill-defined process within the company (1)
- The company's lack of understanding of the benefits of partnering with patient groups (1)
- Company(s) has not been able to offer meaningful or useful input (1)
- Lack of transparency or openness in communication on the part of the company (1)

PG_INDBARR In your opinion, what is the most important barrier to engaging with Industry as it relates to clinical trials?

- Unsure of who to contact in the company (1)
- Lack of clarity in roles (2)
- Mistrust in regards to the company's motives (8)
- Company's reluctance to share data (9)
- Intellectual property issues (10)
- Other (11) ____________________
- Don't know (7)
- Lack of clarity in expectations or anticipated outcomes (6)
- Unsure what value our organization brings to the company's researchers (3)
- Companies are not interested in engaging with us (4)
- Companies do not listen to our perspectives (5)

PG_CTDSUPP Has your organization supported clinical trial development in any of these ways? (Please choose all that apply)

- A research agenda focused on clinical trials and infrastructure (1)
- We develop internal policies and procedures for working with research sponsors (1)
- We write or support the development of handbooks or manuals for clinical trial operations (1)
- We support the development of training manuals for investigators or site personnel (1)
- We participate in investigator or site performance evaluation forms (1)
- Other (1) ____________________
- Our organization does not employ any of the tools listed above to support clinical trials (1)
- A research agenda focused on translational research (1)

PG_COMPLET Congratulations - you have completed the survey!   Your answers are extremely important to us and your efforts are greatly appreciated.   PLEASE NOTE! None of your answers will be recorded until you click the 'NEXT' button below.  If you would like to go back at this time to change any answers, you may do so now.  Otherwise, to record your final answers, you must click the NEXT button.

OTHER   To be eligible for this study, you must be affiliated with a company, academic institution, or patient group.   Your response indicated that you are not eligible for our study.   Thank you for your interest!
