# Supplementary material for: Patient Engagement Practices in Clinical Research among Patient Groups, Industry, and Academia in the United States: A Survey
Source: PLoS One. 2015 Oct 14;10(10):e0140232. doi: 10.1371/journal.pone.0140232 (PMC4605726; doi:10.1371/journal.pone.0140232)
Supplement: S1 Dataset — (DOCX) [file pone.0140232.s002.docx]

S1 Dataset available at the CTTI website (<http://www.ctti-clinicaltrials.org/what-we-do/investigational-plan/patient-groups>).
